# Host plant range of a fruit fly community (Diptera: Tephritidae): does fruit composition influence larval performance?

**DOI:** 10.1186/s12898-016-0094-8

**Published:** 2016-09-20

**Authors:** Abir Hafsi, Benoit Facon, Virginie Ravigné, Frédéric Chiroleu, Serge Quilici, Brahim Chermiti, Pierre-François Duyck

**Affiliations:** 1 CIRAD, UMR PVBMT, 97410 Saint Pierre, France; 2 Institut Supérieur Agronomique de Chott-Mariem, Laboratoire d’Entomologie et de Lutte Biologique, Université de Sousse, 4042 Sousse, Tunisia; 3 UMR « Centre de Biologie pour la Gestion des Populations », INRA-SPE, 755 avenue du Campus, Agropolis, CS 30016, 34988 Montferrier sur Lez, Cedex, France; 4 UMR « Peuplements Végétaux et Bio-agresseurs en Milieu Tropical », CIRAD Pôle de Protection des Plantes, 7 chemin de l’Irat, 97410 Saint Pierre, La Réunion France

**Keywords:** Tephritidae, Nutriments composition, Carbohydrate, Food webs, Host range

## Abstract

**Background:**

Phytophagous insects differ in their degree of specialisation on host plants, and range from strictly monophagous species that can develop on only one host plant to extremely polyphagous species that can develop on hundreds of plant species in many families. Nutritional compounds in host fruits affect several larval traits that may be related to adult fitness. In this study, we determined the relationship between fruit nutrient composition and the degree of host specialisation of seven of the eight tephritid species present in La Réunion; these species are known to have very different host ranges *in natura*. In the laboratory, larval survival, larval developmental time, and pupal weight were assessed on 22 fruit species occurring in La Réunion. In addition, data on fruit nutritional composition were obtained from existing databases.

**Results:**

For each tephritid, the three larval traits were significantly affected by fruit species and the effects of fruits on larval traits differed among tephritids. As expected, the polyphagous species *Bactrocera zonata*, *Ceratitis catoirii*, *C. rosa*, and *C. capitata* were able to survive on a larger range of fruits than the oligophagous species *Zeugodacus cucurbitae*, *Dacus demmerezi*, and *Neoceratitis cyanescens*. Pupal weight was positively correlated with larval survival and was negatively correlated with developmental time for polyphagous species. Canonical correspondence analysis of the relationship between fruit nutrient composition and tephritid survival showed that polyphagous species survived better than oligophagous ones in fruits containing higher concentrations of carbohydrate, fibre, and lipid.

**Conclusion:**

Nutrient composition of host fruit at least partly explains the suitability of host fruits for larvae. Completed with female preferences experiments these results will increase our understanding of factors affecting tephritid host range.

**Electronic supplementary material:**

The online version of this article (doi:10.1186/s12898-016-0094-8) contains supplementary material, which is available to authorized users.

## Background

Arthropods constitute the most diverse group of animals on Earth, and a large fraction of Arthropod species are phytophagous [1]. Phytophagous insects range from those that are strictly monophagous, i.e., that are able to develop on only one host plant, to those that are extremely polyphagous, i.e., that are able to develop on hundreds of plant species belonging to numerous families [2]. Most phytophagous insect species, however, are specialised for feeding on a small range of host plants, and this specialisation is thought to have contributed to the huge diversification of insects that consume plants [1, 3, 4].

The diet breadth of phytophagous insect is shaped by many evolutionary and ecological processes [3, 5, 6]. In most species, females locate, recognise and accept host plants for oviposition, eggs hatch on the host plants, and larvae develop to the adult stage by consuming various parts of the host plants. Diet breadth is therefore the product of female choice and larval performance [1, 7]. Both traits have been shown to evolve rather rapidly [8]. As a result of their joint evolution, female preferences generally but not always correlate with larval performances [9]. The lack of correlation between female preference and larval performance has been previously reported in some studies [10–13]. In particular, the link between female preference and larval performance varies with the degree of diet specialization [6] and depends on the taxonomic diversity of studied host plants [6, 12]. Correlations between female preference and larval performance are more often observed for monophagous insects than for polygophagous insects and across and within plant families than among genotypes within a plant species [6, 12]. Balgawi et al. [10] suggested that host preferences may not be the result of optimization of the preference-performance relationship but linked with other behavioural, physio-chemical and/or physiological associations between insects and their larval hosts. In the field, availability and abundance of host plants and predation may also modify this relationship [13, 14]. While females very rarely prefer a plant that does not support good performance of their offspring [6], larvae may often develop on a larger set of host plants than that selected by females [11, 15, 16]. Hence, larval performance can provide insight into the potential diet breadth of a species [17].

Larval performance is influenced by a number of factors related to the ecological context, i.e., which plants are present in the environment and their availability for insects [18, 19], and to plant intrinsic value for larval development and survival [10, 20–22]. Plants furnish phytophagous insects with an array of vital resources. Moreover, plants in most families contain toxic compounds, including secondary metabolites [23, 24]. Secondary metabolites have been widely described in the vast literature on insect-plant coevolution [25–27]. Chemicals can alter the nutritional value of plant tissues by making them poisonous [28, 29] especially to polyphagous species that feed on vegetative organs or inflorescences. Some polyphagous insects, however, feed and develop in the pulp of fleshy fruits from a wide range of species and families. Frugivorous Tephritidae, for example, feed on many kinds of ripening and commercially grown fruits whose defensive compounds disappear when fruits are ripe [30].

Various nutritional compounds in host plants affect the life history traits of phytophagous larvae [31, 32]. Carbohydrates and lipids in particular greatly affect larval performance, while mineral nutrition may be more crucial for adult fecundity [25]. As a consequence, plants that differ in nutrient content often differ in their effects on insect fitness [21, 22, 26]. Understanding how larval performance relates to plant nutrient composition should therefore shed light on the determinants of insect host range.

Plant availability for insects depends on plant abundance and phenology but also on biotic interactions. In most if not all natural or agronomic landscapes, the same plants can serve as hosts for several phytophagous insect species, whose host ranges may overlap at different scales [33, 34]. Understanding how phytophagous insect communities organize on a given plant community requires the study of the determinants of diet breadth for the different insect species that share a large part of their host range in a given environment.

The tephritids of La Réunion Island are ideal for studying the effects of fruit composition on a community of phytophagous insects. The tephritids, also called true fruit flies, are economically important because many species in this family attack important fruit and vegetable crops in tropical and subtropical regions worldwide. La Réunion hosts eight tephritid species (two indigenous and six exotic) that infest a large number of cultivated and wild host plants in the same, rather small area; the island occupies 2512 km^2^. These eight species differ in diet breadth. Four species (*Ceratitis catoirii*, *C. capitata*, *C. rosa*, and *Bactrocera zonata*) are polyphagous [35]. *Ceratitis rosa*, for example, is found on 60 host species belonging to 20 families in La Réunion [36]. In contrast, the four other species are considered to be oligophagous, i.e., they have a limited host range; *Dacus demmerezi*, *D. ciliatus*, and *Zeugodacus cucurbitae* are found mostly on Cucurbitaceae, and *Neoceratitis cyanescens* is mostly associated with Solanaceae [36]. In spite of these important differences, the diet breadths of the eight species do overlap in that the polyphagous species are able to infest Cucurbitaceae or Solanaceae and the oligophagous species may infest some hosts belonging to families other than the Cucurbitaceae or Solanaceae.

Laboratory studies with *C. catoirii*, *C. capitata*, *C. rosa*, and *B. zonata* [37] and other studies with the tephritids *B. dorsalis* [38] and *C. fasciventris* [39] have indicated that larval performances can differ drastically on different host species. The nutrient content of a larval diet greatly affects larval growth, developmental time, and survival and also affects the number and the fitness of the adult fruit flies produced [38, 40–42]. For instance, the low concentrations of carbohydrate in the upper parts of papaya and orange fruits reduce the development of *C. capitata*, while the high concentrations in the lower parts of the fruits enhance larval development [43]. Similarly, Nash and Chapman [44] found that *C. capitata* developmental time and larval survival are affected by protein quantity and quality as well as by carbohydrate quantity and quality.

The studies cited in the previous paragraph suggested that fruit nutrient composition may be an important factor influencing the organization of tephritid communities. Determining whether this is true requires basic information on the relationship between fruit nutrient composition and the performance of individual tephritid species. In this study, we documented the potential host range of seven of the eight tephritid species in La Réunion by measuring larval performance on 22 host plants belonging to 11 plant families (Table 1), representing the most common crops and wild plants affected by tephritids on the island. For these 22 host plants, we gathered information on fruit nutrient composition from the literature and examined the extent to which fruit biochemical composition was associated with the observed differences in tephritid larval performance.

**Table 1:** List of host species studied

Family	Species	Common name
Anacardiaceae	*Mangifera indica*	Mango
Annonaceae	*Annona reticulata*	Custard apple
Caricaceae	*Carica papaya*	Papaya
Combretaceae	*Terminalia catappa*	Indian almond
Cucurbitaceae	*Citrullus lanatus*	Water melon
	*Cucumis melo*	Melon
	*Cucumis sativus*	Cucumber
	*Cucurbita maxima*	Pumpkin
	*Cucurbita pepo*	Zucchini
	*Sechium edule*	Chayote
Moraceae	*Ficus carica*	Fig
Myrtaceae	*Psidium cattleyanum*	Strawberry guava
	*Psidium guajava*	Guava
Oxalidaceae	*Averrhoa carambola*	Star fruit
Rosaceae	*Eriobotrya japonica*	Loquat
	*Prunus domestica*	Plum
	*Prunus persica*	Peach
Rutaceae	*Citrus reticulata Blanco*	Mandarin
Solanaceae	*Capsicum frutescens*	Chilli
	*Solanum betacea*	Tree tomato
	*Solanum lycopersicum*	Tomato
	*Solanum melongena*	Eggplant

## Methods

### Tephritids

This study of tephritids in La Réunion included four polyphagous species (*C. catoirii*, *C. capitata*, *C. rosa*, and *B. zonata*) and three oligophagous species (*D. demmerezi* and *Z. cucurbitae* are mostly associated with cucurbit hosts, and *N. cyanescens* is mostly associated with Solanaceae fruits). *Dacus ciliatus* could not be included because of difficulties in its rearing. The seven species were reared from specimens initially obtained from rose-apple (*Syzygium jambos*) for *C. catoirii*, beach naupaka (*Scaevola taccada*) for *C. capitata*, rose-apple for *C. rosa*, Indian almond (*Terminalia catappa*) for *B. zonata*, cucumber (*Cucumis sativus*) for *D. demmerezi*, zucchini (*Cucurbita pepo*) for *Z. cucurbitae*, and bugweed (*Solanum mauritianum*) for *N. cyanescens*. No permissions were required to collect these samples from the field. Larvae of *C. catoirii*, *C. capitata*, *C. rosa*, and *B. zonata* were subsequently reared on an artificial diet [45, 46] for generations F147–F160, F4–F10, F1–F3, and F118–F131, respectively. Larvae of *D. demmerezi* and *Z. cucurbitae* were reared on zucchini for generations F10–F18 and F54–F69, respectively, and larvae of *N. cyanescens* were reared on potato (*Solanum tuberosum*) for generations F10–F18. The species were not reared on the same food substrate because each was placed on the adequate artificial diet or host plant that enabled optimal development under laboratory conditions. In addition, some of these species are difficult to find in the field or to maintain in the laboratory, which explains the variability in the numbers of generations among species. Populations were maintained at several thousands of individuals per generation. Laboratory rearing was conducted under constant environmental conditions (25 ± 1 °C; 70 % relative humidity; L:D 12:12 photoperiod supplemented with natural light to maintain twilight conditions). Eggs were collected using an inverted perforated plastic cup swabbed with the flesh of host fruits; citrus for *C. catoirii*, *C. capitata*, *C. rosa*, and *B. zonata*; pumpkin for *D. demmerezi* and *Z. cucurbitae*; and potato for *N. cyanescens*. Eggs were placed in a 2 % Nipagine/sodium benzoate solution and were incubated in environmental chambers at a constant temperature of 25 °C for 30 h for *Z. cucurbitae*, 55 h for *N. cyanescens*, and for 48 h for the other five species.

### Experimental setup

An important technical challenge in measuring larval performance on intact fruits of many different plant species is the heterogeneity in fruit decay. We developed simplified diets that contained 250 g of fruit pulp without peel or seeds, 4 g of agar–agar (to provide a suitable texture), and 10 ml of a 4 % Nipagine/sodium benzoate solution (to prevent fungal and bacterial growth). While these diets differed from fresh fruits in term of physical texture, they allowed measuring individual fitness traits, following a high number of homogenous replicates, and obtaining comparable measurements of larval performance with a focus on fruit nutrient value.

Components of the diet were blended and placed in individual 5-ml plastic cups, each containing 5 g of mixture. Each combination of tephritid species and fruit species was represented by 50 replicate cups, giving a total of 7700 cups. One neonate larva (<2 h old) was placed in each cup. Each cup was set in a shallow pan with sand to allow pupation. Because temperature greatly affects tephritid larval development, all experiments were carried out in environmental chambers at a constant temperature of 25 °C, which is favourable for the seven species [45–48].

### Larval performance

Three indicators of larval performance were assessed: survival rate, developmental time, and pupal weight. Pupal weight is a good indicator of female fecundity and therefore of individual female fitness in Tephritidae [49, 50]. Every 48 h during 60 days, all cups were examined and pupae were collected. Larval survival was recorded as the number of pupae recovered from each host. Developmental time was recorded as the time from placement in the cup to pupation. Each pupa was weighed (Sartorius® Germany, precision: 10^−4^ g).

### Fruit nutrient composition

To study the relationship between tephritid larval performance and fruit nutrient composition, we gathered data on nutrient composition from the United States Department of Agriculture (USDA) National Nutrient Database for Standard Reference, the ANSES (French Agency for Food, Environmental and Occupational Health Safety) French Food Composition Table, and published studies (data used and references are presented in Additional file 1). For the 22 plant species, data on the content of water, proteins, total carbohydrates, fat, fibres, sodium, magnesium, potassium, phosphorus, calcium, iron, and vitamin C are presented in a data set available in Additional file 1. Nutrients that could not be documented for all 22 plant species were excluded from the analysis to avoid problems caused by missing data. While nutrient content obtained from the literature probably did not strongly differ from the diets used for trait measurements, water content could have been more affected during the processing of diets. In order to check if water content in database and diets were representative of fresh fruits, we measured water contents in diets and fresh fruits for six fruit species (*Annona reticulate*, *Carica papaya*, *Cucumis sativus*, *Cucurbita maxima*, *Psidium gujava*, and *Solanum lycopersicum*). A strong linear relationship was observed between the water contents of artificial diets and the water contents estimates from database (R^2^ = 0.87; P < 0.001; Y = 0.90X + 12.13) and between water contents in fresh fruits and water contents in artificial diets (P < 0.0001; R^2^ = 0.97, Y = 0.96X + 6.28) (Additional file 1). While artificial diets tend to contain more water than expected from literature data (intercept different from 0), differences between fruits were conserved (slope close to 1).

### Statistical analyses

All analyses were performed with R-3.2.2-win [51]. Larval survival was analysed using a general linear model (GLM) with a binomial error as a function of plant species, tephritid species, and interaction. Developmental time and pupal weight were analysed by analysis of variance (ANOVA) as a function of plant species, tephritid species, and interaction. The relationship between the three indicators of larval performance was investigated using linear models.

Canonical correspondence analysis (CCA) [52] was used to study the relationship between nutrient composition of the 22 host fruits and larval survival of the seven tephritid species using the function *pcaiv* in the *ade4* package [53]. The CCA analysis consist of carrying out a factorial correspondence analysis (FCA) on the fitted variables (larval survival of seven tephritid species on 22 fruit species) after the regression on the instrumental variables (chemical composition of 22 fruit species). The significance of CCA was tested by a Monte-Carlo test [54] that evaluated the significance and the stability of decomposition of the total inertia of larval survival with only permutation of the rows of biochemical composition table.

Results of CCA analyses (presented in Additional file 2) showed that the first axis of the factorial space explained 64.5 % of the global variability. The variability in larval survival of the seven tephritid species was mostly explained (64.9 %) by the contents of 12 nutrients in the 22 fruit species. A Monte-Carlo test showed that the inertia projected by 1000 permutations was not significant (P = 0.141). Water, lipid, carbohydrate, and fibre were the nutrients most correlated with the first axis (correlations >0.5). A second CCA analysis restricted to these four nutrients was carried out to clarify their influence on larval survival and to increase the stability of the decomposition of inertia.

## Results

### Influence of host fruit on larval survival

**Fig. 1:**
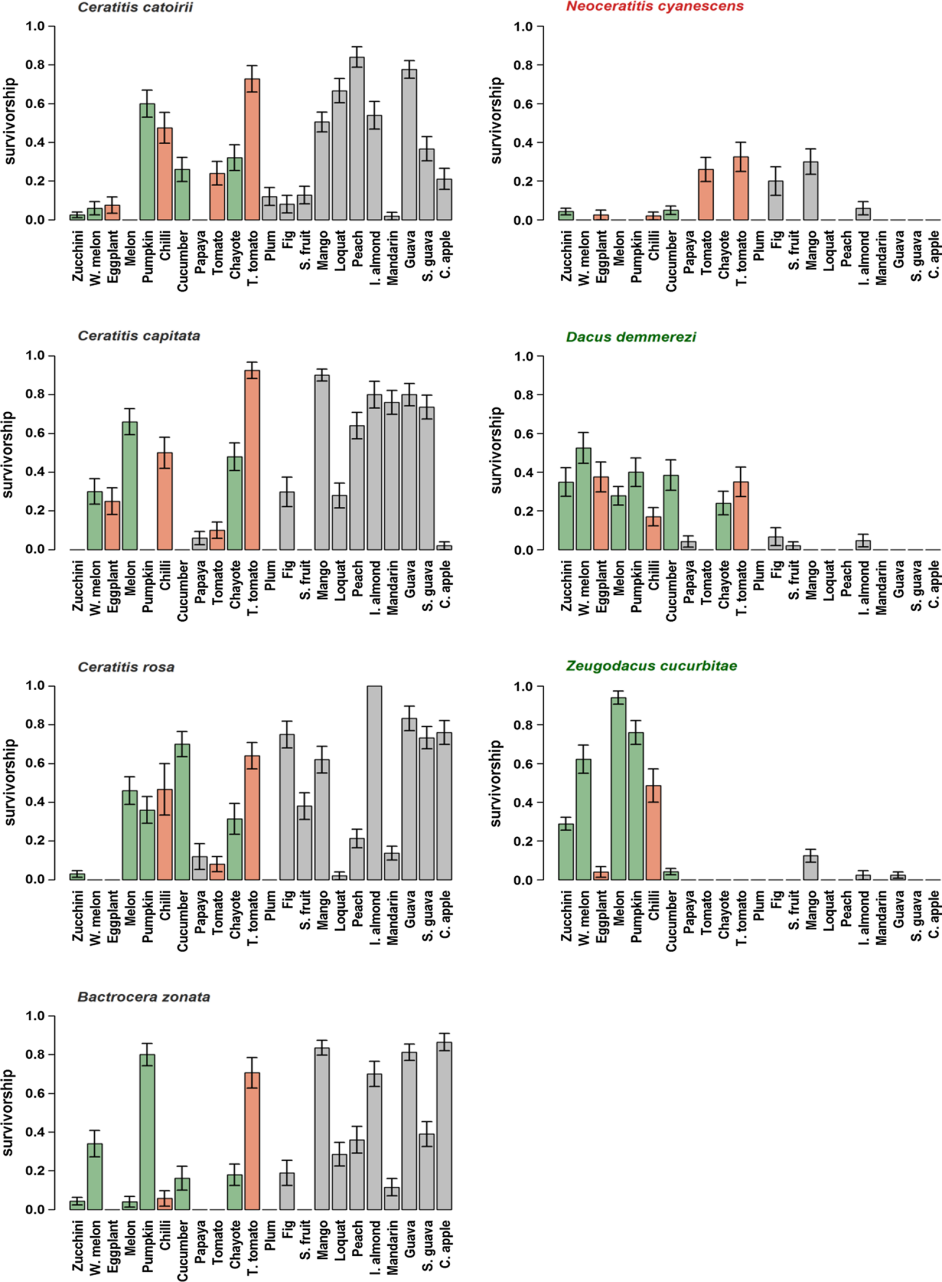
Larval survival for seven tephritid species reared on 22 host fruit species occurring in La Reunion. Values are mean ± SE. Host fruits belonging to the Cucurbitaceae and Solanaceae are indicated by *green* and *red*, respectively. Host fruits are ordered by coordinate of the first axis of CCA analyses (see “Methods” section)

Larval survival rates differed significantly among the fruit species (ΔDev_21, 1119_ = 7948, P < 0.001) and among the tephritid species (ΔDev_6, 639_ = 7942, P < 0.001); the interaction between tephritid and fruit species was also significant (ΔDev_125, 2120_ = 7817, P < 0.001) (Fig. 1). Larvae of the polyphagous species *B. zonata*, *C. catoirii*, *C. capitata*, and *C. rosa* survived on a wide range of hosts (on 17–20 of the 22 fruit species tested). On these host fruits, the four polyphagous tephritid species had a larval survival rate ranging from 2 to 100 %, with the highest larval survival exceeding 60 % in tree tomato, guava, mango, and Indian almond. *Dacus demmerezi* and *Z. cucurbitae*, which are considered specialised on Cucurbitaceae fruits, survived on 13 and 10 fruit species, respectively; survival of *D. demmerezi* was highest on six of the cucurbit species, and survival of *Z. cucurbitae* was highest on five of the cucurbit species. *Dacus demmerezi* and *Z. cucurbitae* also survived on fruits from the Solanaceae (eggplant and chilli), the Anacardiaceae (mango), and the Combretaceae (Indian almond). *Neoceratitis cyanescens*, which is considered specialised on the Solanaceae family, had the highest survival rate on fruit species belonging to the Solanaceae but also on mango, and was able to survive, albeit with lower survival rates, on cucumber and zucchini fruits.

### Influence of host fruit on pupal weight

**Fig. 2:**
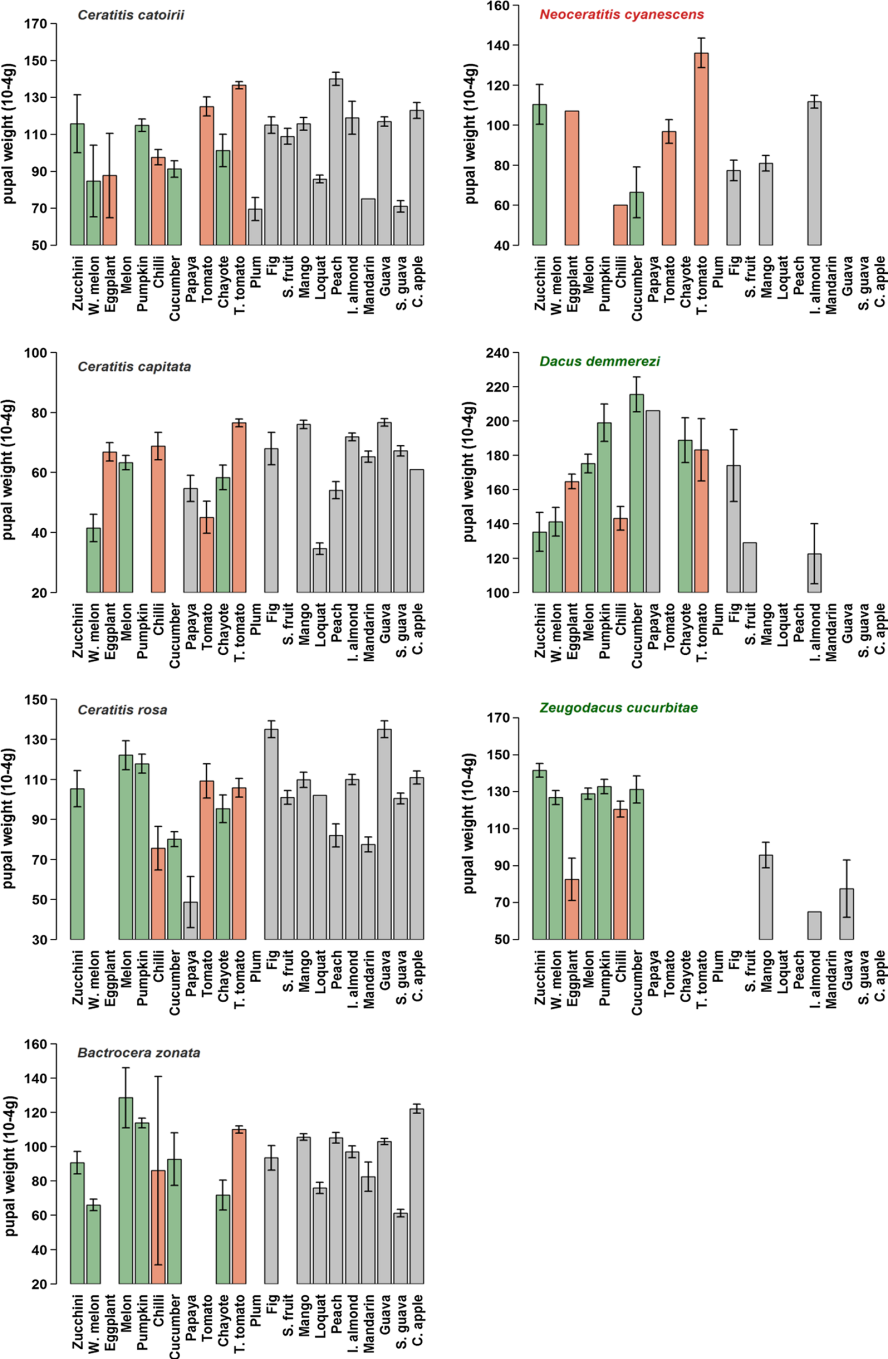
Pupal weight (10^−4^ g) for the seven tephritid species reared on 22 host fruit species occurring in La Reunion. Values are mean ± SE. Host fruits belonging to the Cucurbitaceae and Solanaceae are indicated by *green* and *red*, respectively. Host fruits are ordered by coordinate of the first axis of CCA analyses (see “Methods” section). Pupal weight scales among species were changed for better representation

Pupal weight differed significantly among fruit species (F_21, 24593_ = 51.1, P < 0.001) and among tephritid species (F_6, 198010_ = 418.7, P < 0.001); the interaction between tephritid and fruit species was also significant (F_76, 4457_ = 9.4, P < 0.001) (Fig. 2). Across fruit species, pupal weight was lowest for *C. capitata* and highest for *D. demmerezi*. For the four polyphagous tephritids, pupal weight was highest on peach and tree tomato for *C. catoirii*; on guava, mango, and tree tomato for *C. capitata*; on guava for *C. rosa*; and on custard apple for *B. zonata*. For the three oligophagous tephritids, pupal weight was highest on cucumber for *D. demmerezi*, on zucchini and cucumber for *Z. cucurbitae*, and on tree tomato for *N. cyanescens*.

### Relationship between pupal weight, larval survival, and developmental time

Developmental time differed significantly among fruit species (F_21, 965_ = 31.2, P < 0.001) and among tephritid species (F_6, 6410_ = 207.2, P < 0.001); the interaction between tephritid and fruit species was also significant (F_76, 451_ = 14.6, P < 0.001) (Additional file 3). On all fruits that supported larval survival, pupal weight was positively correlated with larval survival for *C. catoirii* (P < 0.001), *C. capitata* (P < 0.001), *C. rosa* (P = 0.0013), and *B. zonata* (P < 0.001), and was negatively correlated with developmental time for *C. catoirii* (P < 0.001), *C. capitata* (P < 0.001), *B. zonata* (P < 0.001), and *Z. cucurbitae* (P = 0.022) (Table 2; Additional file 4). Similar but statistically insignificant relationships were also observed for the other species.

**Table 2:** Analyses by linear models of pupal weight as a function of larval survival and developmental time for seven Tephritidae species reared on 22 fruit species

Tephritidae species	Effect	Residuals; Df	Estimate	F value	P value
*Ceratitis catoirii*	Survival	1; 393	6.71	86.06	<*0.001*
	DT	1; 393	−2.49	290.38	<*0.001*
*Ceratitis capitata*	Survival	1; 413	30.40	161.40	<*0.001*
	DT	1; 413	−0.45	15.95	<*0.001*
*Ceratitis rosa*	Survival	1; 288	22.91	10.43	*0.0013*
	DT	1; 288	−0.09	0.29	0.5880
*Bactrocera zonata*	Survival	1; 406	24.40	188.58	<*0.001*
	DT	1; 406	−2.27	126.27	<*0.001*
*Neoceratitis cyanescens*	Survival	1; 590	68.42	3.37	0.0714
	DT	1; 590	−0.51	0.99	0.3245
*Dacus demmerezi*	Survival	1; 141	−9.04	0.05	0.8152
	DT	1; 141	−0.15	0.03	0.8568
*Zeugodacus cucurbitae*	Survival	1; 193	5.59	2.35	0.1265
	DT	1; 193	−0.66	5.32	*0.0221*

*DT* developmental time, *Survival* larval survival

Significant effects (P < 0.05) are indicated in italic

### Relationship between fruit nutrient composition and larval survival

CCA analysis showed that the first eigenvalue, which explained 74.33 % of the total variation, was overwhelmingly larger than the second one, which explained only 14.26 % of the total variation. Most of the common structure of the two data matrices is therefore contained in the first axis. Water, lipid, carbohydrate, and fibre explained 30.05 % of the variability in survival of larvae of the seven tephritid species reared on 22 fruit species. A Monte-Carlo test showed significant inertia projected for 1000 permutations (P_value_  = 0.028).

**Fig. 3:**
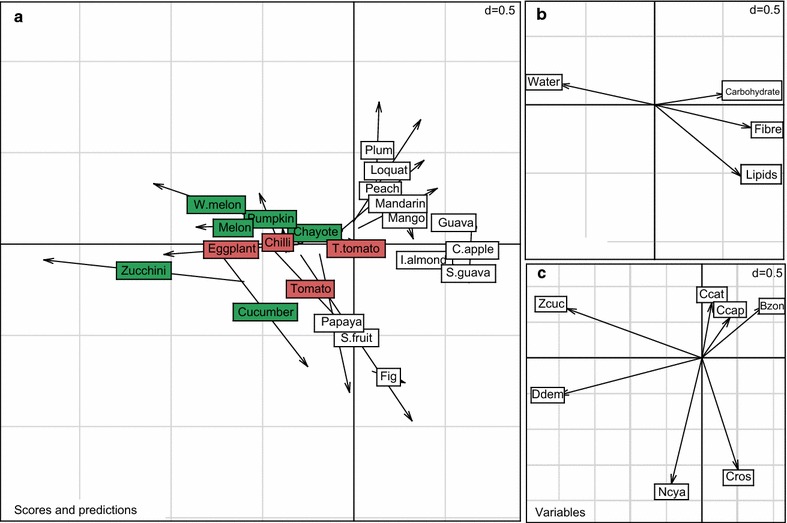
CCA analysis of the relationship between fruit composition and survival of seven tephritid species. **a** Position of the different fruit species in the factorial space of the CCA analysis with contribution of nutrient composition and larval survival on the studied fruits. **b** Contribution of the nutrient composition to the factorial space. **c** Position of larval survival of the seven tephritid species in the factorial space. Host fruits belonging to Cucurbitaceae and Solanaceae families are indicated by *green* and *red*, respectively. Ccat, *C. catoirii*; Ccap, *C. capitata*; Cros, *C. rosa*; Bzon, *B. zonata*; Zcuc, *Z. cucurbitae*; Ddem, *D. demmerezi*; Ncya, *N. cyanescens*

CCA analysis (Fig. 3a) showed that all tested fruits fell into two distinct groups, with species in the Solanaceae (tomato, tree tomato, eggplant, and chili) and Cucurbitaceae (pumpkin, zucchini, cucumber, and water melon) in the first group, and the other species in the second group. Shorter arrows (like those for zucchini, melon, guava, mango, Indian almond, and strawberry guava) indicate a better concordance between fruit nutrient composition and survival of tephritid species larvae.

The direction of the vector for water concentration was opposite to the direction for the vectors for carbohydrate, fibre, and lipid concentration (Fig. 3b), which indicated a negative correlation between these components. The length of these vectors indicates the importance of the components in explaining the structure of the two data matrices; importance decreases with vector length. Vectors of larval survival of *D. demmerezi* and *Z. cucurbitae* (Fig. 3c) were pointed in the same direction as the vector for water and in the opposite direction as the vectors for carbohydrate, fibre, and lipid (Fig. 3b), indicating that survival of *D. demmerezi* and *Z. cucurbitae* larvae was positively correlated with the concentration of water in fruits and was negatively correlated with the concentration of carbohydrate, fibre, and lipid in fruits. Larval survival of *B. zonata*, *C. catoirii*, *C. capitata*, and *C. rosa* was positively correlated with the concentration of carbohydrate, fibre, and lipid in fruits and negatively correlated with the concentration of water in fruits. Larval survival of *N. cyanescens* depended slightly on the components represented by the first axis of the CCA analyses, and was positively correlated with concentration of water and lipid in host fruits.

## Discussion

The present study aimed to evaluate the contribution of available host plants to the performance of tephritid larvae occurring in sympatry. We established that host identity had a marked influence on larval survival, developmental time, and pupal weight. In general, these three fitness traits were positively correlated with each other, i.e., larvae reared on fruits supporting high survival had high pupal weights and short developmental times. More specifically, pupal weight was positively correlated with larval survival for *B. zonata*, *C. catoirii*, *C. capitata*, and *C. rosa* and was negatively correlated with developmental time for *B. zonata, C. catoirii*, *C. capitata*, and *Z. cucurbitae.* Similar relationships between the three traits were also observed for *D. demmerezi* and *N. cyanescens* but at a non-significant level, probably because the statistical power was limited by the low number of host fruits that supported survival of these oligophagous species. Pupal weight has previously been shown to be positively correlated with female fecundity in several tephritids [37, 40, 49]. This absence of trade-offs between fitness components suggests that hosts differ in nutritional value for tephritid development [55]. Some fruits have sufficient nutritional value to maximize all three of the measured performance traits. This is the case for guava, mango, and Indian almond, which supported relatively high survival (up to 60 %) and high pupal weights for the four polyphagous tephritids. In other geographical areas, these fruit species are the most utilized by the *Bactrocera dorsalis* complex of fruit flies [56]. Interestingly, the host range measured in the laboratory for the oligophagous tephritids like *Z. cucurbitae* included fruit species that are not hosts in the field in La Réunion but are hosts in other areas. *Zeugodacus cucurbitae* has been reported to cause damage on mango fruit in Africa and on papaya fruit in India [57, 58], confirming that *Z. cucurbitae* may use these plants in other ecological contexts.

Larval performance measured in tephritid colonies reared in laboratory for several generations may represent an important limitation of this study. Reports of differences between laboratory reared and wild insect strains and populations have gained much attention because of the relevance of such changes in behaviour and life history traits of phytophagous insects [59–62]. However, female oviposition preferences are generally found to change more rapidly than larval performance that is considered more conservative. For example in the bruchid beetle, after 11 generations of natural and artificial selection, genetic changes occurred in the behavioural response to host change, but not in the physiological performance of larvae [63]. Our study has other important limitations. First, we did not assess some potentially important components of specialisation. Female choice, for example, was not measured but can greatly affect fruit fly abundance [13] and represent an important factor that facilitates host-plant adaptation [64]. Second, while symbiotic bacteria in the insect gut can impact larval development and adult fitness [65], these effects were not measured in our study. Symbiotic bacteria assemblages play a major role in detoxification processes thus making plant tissues edible for phytophagous insects and promote adaptation between phytophagous insects and host plants [66, 67]. Finally, we did not assess interactions among fruit fly species. Intra- and interspecific competition among larvae of different species within fruits and between adult females for egg laying sites are potentially important factors affecting host plant use by phytophagous insects in the field [68, 69].So additional research is needed to determine how the results of current study relate to community dynamics.

Multivariate analyses (CCA) indicated that the water, lipid, carbohydrate, and fibre content of fruits may explain 30 % of the variability in the larval performance of the seven tephritid species. Larval survival of the polyphagous species *B. zonata*, *C. catoirii*, *C. capitata*, and *C. rosa* was positively correlated with carbohydrate, lipid, and fibre contents and negatively correlated with water content. In contrast, larval survival of the two species *D. demmerezi* and *Z. cucurbitae* associated with cucurbit hosts was positively correlated with water content and negatively correlated with carbohydrate and lipid content. Larval survival of *N. cyanescens*, which was the one species associated with Solanaceae fruits, was positively correlated with water and lipid contents.

Carbohydrates were previously identified as potentially important determinants of fruit nutritive value for tephritid larvae. Zucoloto [70], and Fernandes Da Silva and Zucoloto [43] showed that larvae of *C. capitata* moved to the part of the fruit containing the highest quantities of carbohydrate. These and current results are consistent with those reported for *C. capitata* [44], Acrididae [71], Drophila [72], and Noctuidae [73].

Behmer [74] suggested that the larvae of some phytophagous insect species prefer high-carbohydrate diets whereas others prefer high-protein diets. Our results suggest that polyphagous tephritids differ from oligophagous tephritids in terms of nutrient requirements. The performance of polyphagous species was strongly associated with carbohydrate, lipid, and fibre contents and was not associated with protein content. This contradicts previous experimental findings that decreases in protein quantity and quality reduced larval development and survival for *C. capitata* [44]. An explanation may be that the protein concentration of host fruit is generally low and invariant among fruits, whereas carbohydrate concentration in fruits is higher and more variable [75]. Carbohydrate concentration but not protein content was retained in our canonical correspondence analysis. In contrast to polyphagous species, the oligophagous tephritids *D. demmerezi* and *Z. cucurbitae* performed better on fruits with high water content than on fruits with high carbohydrate, fibre, and lipid contents. The results of this study are consistent with the hypothesis that polyphagous and oligophagous insects differ in their nutritional requirements [76]. Because pulpy fruits have less structural diversity than stems, leaves, or inflorescences, fruit-feeding tephritids have better prospects to evolve wide host ranges than those tephritids that feed on other plant parts [77].

In the current study, we used those nutrients whose contents in all 22 of the studied fruits were available in published data bases. It follows that some chemical compounds were not included. While nutritional quality of fruit pulp of the plant greatly influences larval performance, host specialisation often cannot be predicted solely by classical nutritional measures. It is also affected by defensive chemicals (secondary metabolites and volatile compounds) and plant physical characteristics (fruit and peel texture) [13, 78, 79]. Plant secondary metabolites are commonly thought to directly or indirectly deter the fecundity or the oviposition of phytophagous insects by being toxic or by reducing nutrient assimilation [25]. Erbout et al. [80] found that larvae of the polyphagous tephritid *C. fasciventris* did not survive in fruits containing high alkaloid concentrations. Among insect herbivores, oligophagous species are less affected than polyphagous species by defensive chemicals in the tissues that they typically consume [81]. This is well illustrated by *N. cyanescens*, which is the only tephritid in La Réunion Island able to survive on the fruits of most species belonging to the Solanaceae; such fruits often contain toxic compounds [49]. Volatile compounds of the fruit peel may determine the attractivity or non-attractivity of host plants to Tephritidae, and thus enable flies to discriminate between host and non-host plants [13, 82]. Hardness of the pericarp/peel determine the ability of the female to ovipositor [79, 83]. While physical and chemical properties of the peel may also affect larval survival, this was not taken into account in our study. For example early instar larvae may suffer from heavy mortality as a consequence of flavedo essential oils and gum secretion in *Citrus* [84–86], of the formation of hardened calluses around egg cavities and of peel mechanical resistance that prevent larvae to reach the fruit pulp [84].

## Conclusions

Our results suggest that nutrient composition at least partly explains the suitability of host fruits for larvae of the seven tephritids in La Réunion Island. From an applied perspective, information on the performance of phytophagous larvae on potential hosts is essential for predicting future host range expansion, population size, and plant damage [32, 87]. Future studies should also investigate female preference to increase our understanding of the factors driving tephritid host range.

## Additional files

10.1186/s12898-016-0094-8 Nutrient contents obtained from the literature for 22 fruit species (g or mg per 100 g of pulp).
10.1186/s12898-016-0094-8 CCA analysis of the relationship between fruit composition and survival of the larvae of seven tephritid species. (a) Position of the fruit species in the factorial space of CCA analyses with contribution of nutrient composition and larval survival on the studied fruits. (b) Contribution of the nutrient composition to the factorial space. (c) Position of larval survival of the seven tephritid species in the factorial space. Host fruits belonging to the Cucurbitaceae and Solanaceae families are in green and red, respectively. Ccat: *C. catoirii*, Ccap: *C. capitata*, Cros: *C. rosa*, Bzon: *B. zonata*, Zcuc : *Z. cucurbitae*, Ddem: *D. demmerezi*, Ncya: *N. cyanescens.*

10.1186/s12898-016-0094-8 Duration (days) of the larval stage of seven tephritid species reared on 22 different host fruits occurring in La Reunion. Values are means ± SE. Host fruits belonging to the Cucurbitaceae and Solanaceae families are in green and red, respectively. Host fruits are ordered by coordinate of the first axis of the CCA analyses (see “Methods” section).
10.1186/s12898-016-0094-8 Relationship between pupal weight (10^−4^ g) and larval duration (days) for seven tephritid species reared on 22 different host fruits. Determination coefficients (R^2^) and regression lines are given when relationships are significant (P< 0.05).

## Abbreviations

USDA: United States Department of Agriculture
ANSES: French Agency for Food, Environmental and Occupational Health Safety
CCA: canonical correspondence analysis
ANOVA: analysis of variance
GLM: general linear model

## References

[1] Jaenike J (1990). Host specialization in phytophagous insects. Annu Rev Ecol Syst.

[2] Jermy T (1984). Evolution of insect-host plant relationships. Am Nat..

[3] Futuyma DJ, Moreno G (1988). The evolution of ecological specialization. Annu Rev Ecol Syst.

[4] Schoonhoven LM, Jermy T, van Loon JJA (1998). Insect-plant biology: from physiology to evolution.

[5] Abrams PA (2006). Adaptive change in the resource-exploitation traits of a generalist consumer: the evolution and coexistence of generalists and specialists. Evolution.

[6] Gripenberg S, Mayhew PJ, Parnell M, Roslin T (2010). A meta-analysis of preference-performance relationships in phytophagous insects. Ecol Lett.

[7] Ravigné V, Dieckmann U, Olivieri I (2009). Live where you thrive: joint evolution of habitat choice and local adaptation facilitates specialization and promotes diversity. Am Nat.

[8] Craig TP, Itami JK, Tilmon KJ (2008). Evolution of preference and performance relationships. Specialization, speciation, and radiation The evolutionary biology of herbivorous insects.

[9] Keeler MS, Chew FS (2008). Escaping an evolutionary trap: preference and performance of a native insect on an exotic invasive host. Oecologia.

[10] Wiklund C (1973). Host plant suitability and the mechanism of host selection in larvae of *Papilio machaon*. Entomol Exp Appl.

[11] Thompson JN (1988). Evolutionary ecology of the relationship between oviposition preference and performance of offspring in phytophagous insects. Entomol Exp Appl.

[12] Balagawi S, Drew RA, Clarke AR (2013). Simultaneous tests of the preference-performance and phylogenetic conservatism hypotheses: is either theory useful?. Arth Plant Int..

[13] Fitt GP (1986). The roles of adult and larval specialisations in limiting the occurrence of five species of *Dacus* (Diptera: Tephritidae) in cultivated fruits. Oecologia.

[14] Cunningham J (2012). Can mechanism help explain insect host choice?. J Evol Biol.

[15] Mayhew PJ (1997). Adaptive patterns of host-plant selection by phytophagous insects. Oikos.

[16] Price PW, Leather SR, Watt AD, Mills NJ, Walters KFA (1994). Patterns in the population dynamics of insect herbivores. Individuals, populations and patterns in ecology.

[17] Futuyma DJ, Keese MC, Gerald AR, May RB (1992). Evolution and coevolution of plants and phytophagous arthropods. Herbivores their interaction with secondary plant metabolites ecological and evolutionary processes.

[18] Kuussaari M, Singer M, Hanski I (2000). Local specialization and landscape-level influence on host use in an herbivorous insect. Ecology.

[19] Fahrig L, Paloheimo J (1988). Effect of spatial arrangement of habitat patches on local population size. Ecology.

[20] Friberg M, Wiklund C (2009). Host plant preference and performance of the sibling species of butterflies *Leptidea sinapis* and *Leptidea reali*: a test of the trade-off hypothesis for food specialisation. Oecologia.

[21] Murphy SM (2007). The effect of host plant on larval survivorship of the Alaskan swallowtail butterfly (*Papilio machaon aliaska*). Entomol Exp Appl.

[22] Scriber JM, Feeny P (1979). Growth of herbivorous caterpillars in relation to feeding specialization and to the growth form of their food plants. Ecology.

[23] Cornell HV, Hawkins BA (2003). Herbivore responses to plant secondary compounds: a test of phytochemical coevolution theory. Am Nat.

[24] Mithöfer A, Boland W (2012). Plant defense against herbivores: chemical aspects. Annu Rev Plant Biol.

[25] Awmack CS, Leather SR (2002). Host plant quality and fecundity in herbivorous insects. Annu Rev Entomol.

[26] Chapman R, Hardege JD (2009). Foraging and food choice in phytophagous insects. Chemical ecology.

[27] Chapman R (1974). The chemical inhibition of feeding by phytophagous insects: a review. Bull Entomol Res.

[28] Howe GA, Jander G (2008). Plant immunity to insect herbivores. Annu Rev Plant Biol.

[29] Schoonhoven L, Meerman J (1978). Metabolic cost of changes in diet and neutralization of allelochemics. Entomol Exp Appl.

[30] Aluja M, Mangan RL (2008). Fruit fly (Diptera: Tephritidae) host status determination: Critical conceptual, methodological, and regulatory considerations. Annu Rev Entomol.

[31] Bernays E, Chapman R, Bernays E, Chapman R (1994). Host-plant selection by phytophagous insects. Behavior: the process of host-plant selection.

[32] Scriber J, Slansky JF (1981). The nutritional ecology of immature insects. Annu Rev Entomol.

[33] Koerner SE, Burkepile DE, Fynn RW, Burns CE, Eby S, Govender N, Hagenah N, Matchett KJ, Thompson DI, Wilcox KR (2014). Plant community response to loss of large herbivores differs between North American and South African savanna grasslands. Ecology.

[34] Ritchie M, Olff H, Olff H, Brown VK, Drent RH (1999). Herbivore diversity and plant dynamics: compensatory and additive effects. Herbivores: between plants and predators.

[35] White IM, Elson-Harris MM (1992). Fruit flies of economic significance: their identification and bionomics.

[36] Quilici S, Jeuffrault E (2001). Plantes-hôtes des mouches des fruits: Maurice, Réunion, Seychelles.

[37] Duyck PF, David P, Pavoine S, Quilici S (2008). Can host-range allow niche differentiation of invasive polyphagous fruit flies (Diptera: Tephritidae) in La Réunion?. Ecol Entomol.

[38] Ekesi S, Nderitu PW, Chang CL (2007). Adaptation to and small-scale rearing of invasive fruit fly *Bactrocera invadens* (Diptera: Tephritidae) on artificial diet. Ann Entomol Soc Am.

[39] Ekesi S, Mohamed SA, Chang CL (2014). A liquid larval diet for rearing *Bactrocera invadens* and *Ceratitis fasciventris* (Diptera: Tephritidae). Int J Trop Insect Sci.

[40] Krainacker D, Carey J, Vargas R (1987). Effect of larval host on life history traits of the Mediterranean fruit fly, *Ceratitis capitata*. Oecologia..

[41] Vargas R, Mitchell S, Hsu C-L, Walsh WA (1994). Laboratory evaluation of diets of processed corncob, torula yeast, and wheat germ on four developmental stages of Mediterranean fruit fly (Diptera: Tephritidae). J Econ Entomol.

[42] Kaspi R, Mossinson S, Drezner T, Kamensky B, Yuval B (2002). Effects of larval diet on development rates and reproductive maturation of male and female Mediterranean fruit flies. Physiol Entomol..

[43] Fernandes Da Silva PG, Zucoloto FS (1993). The influence of host nutritive value on the performance and food selection in *Ceratitis capitata* (Diptera, Tephritidae). J Insect Physiol.

[44] Nash WJ, Chapman T (2014). Effect of dietary components on larval life history characteristics in the medfly (*Ceratitis capitata*: Diptera, Tephritidae). PLoS ONE.

[45] Duyck PF, Quilici S (2002). Survival and development of different life stages of three Ceratitis spp. (Diptera: Tephritidae) reared at five constant temperatures. Bull Entomol Res.

[46] Duyck PF, David P, Quilici S (2004). A review of relationships between interspecific competition and invasions in fruit flies (Diptera: Tephritidae). Ecol Entomol.

[47] Brévault T, Quilici S (2000). Relationships between temperature, development and survival of different life stages of the tomato fruit fly, *Neoceratitis cyanescens*. Entomol Exp Appl.

[48] Vayssières JF, Carel Y, Coubes M, Duyck PF (2008). Development of immature stages and comparative demography of two cucurbit-attacking fruit flies in Reunion Island: *Bactrocera cucurbitae* and *Dacus ciliatus* (Diptera Tephritidae). Environ Entomol.

[49] Brévault T, Duyck PF, Quilici S (2008). Life-history strategy in an oligophagous tephritid: the tomato fruit fly, *Neoceratitis cyanescens*. Ecol Entomol.

[50] Krainacker D, Carey J, Vargas R (1989). Size-specific survival and fecundity for laboratory strains of two tephritid (Diptera: Tephritidae) species: implications for mass rearing. J Econ Entomol.

[51] R Development Core Team (2008). R: a language and environment for statistical computing.

[52] Ter Braak CJ (1986). Canonical correspondence analysis: a new eigenvector technique for multivariate direct gradient analysis. Ecology.

[53] Dray S, Dufour A-B (2007). The ade4 package: implementing the duality diagram for ecologists. J Stat Softw.

[54] Rubinstein RY, Kroese DP (2011). Simulation and the Monte Carlo method.

[55] Stearns SC (1992). The evolution of life histories.

[56] Clarke AR, Armstrong KF, Carmichael AE, Milne JR, Raghu S, Roderick GK, Yeates DK (2005). Invasive phytophagous pests arising through a recent tropical evolutionary radiation: the *Bactrocera dorsalis* complex of fruit flies. Annu Rev Entomol.

[57] Vayssières JF, Rey JY, Traoré L (2007). Distribution and host plants of *Bactrocera cucurbitae* in West and Central Africa. Fruits.

[58] Kapoor V, Agarwal M, Cavalloro R. Fruit flies and their increasing host plants in India:. In: Proceedings of the CEC/IOBC international symposium on fruit flies of economic importance, Athens, Greece. Rotterdam: AA Balkema, Published; 1982. p. 252–7.

[59] Vargas RI, Carey JR (1989). Comparison of demographic parameters for wild and laboratory-adapted Mediterranean fruit fly (Diptera: Tephritidae). Ann Entomol Soc Am.

[60] Genc H. Adaptation process of wild population of olive fruit fly (*Bactrocera Oleae* (Rossi) (Diptera: Tepritidae)) into the laboratory. In: International conference on biological, civil and environmental engineering (BCEE-2014). Dubai (UAE). Published; 2014.

[61] Vaníčková L, do Nascimento RR, Hoskovec M, Ježková Z, Břízová R, Tomčala A, Kalinová B (2012). Are the wild and laboratory insect populations different in semiochemical emission? The case of the medfly sex pheromone. J Agric Food Chem.

[62] Richerson JV, Cameron EA (1974). Differences in pheromone release and sexual behavior between laboratory-reared and wild gypsy moth adults. Environ Entomol.

[63] Wasserman SS, Futuyma DJ (1981). Evolution of host plant utilization in laboratory populations of the southern cowpea weevil, *Callosobruchus maculatus* Fabricius (Coleoptera: Bruchidae). Evolution.

[64] West-Eberhard MJ (1989). Phenotypic plasticity and the origins of diversity. Annu Rev Ecol Syst.

[65] Augustinos AA, Kyritsis GA, Papadopoulos NT, Abd-Alla AM, Cáceres C, Bourtzis K (2015). Exploitation of the medfly gut microbiota for the enhancement of sterile insect technique: use of *Enterobacter* sp. in larval diet-based probiotic applications. PloS ONE.

[66] Tsuchida T, Koga R, Fukatsu T (2004). Host plant specialization governed by facultative symbiont. Science.

[67] Frago E, Dicke M, Godfray HCJ (2012). Insect symbionts as hidden players in insect—plant interactions. Trends Ecol Evol.

[68] Feder JL, Reynolds K, Go W, Wang EC (1995). Intra-and interspecific competition and host race formation in the apple maggot fly, *Rhagoletis pomonella* (Diptera: Tephritidae). Oecologia.

[69] Duyck PF, David P, Junod G, Brunel C, Dupont R, Quilici S (2006). Importance of competition mechanisms in successive invasions by polyphagous tephritids in La Réunion. Ecology.

[70] Zucoloto FS (1987). Feeding habits of *Ceratitis capitata* (Diptera: Tephritidae): can larvae recognize a nutritionally effective diet?. J Insect Physiol.

[71] Joern A, Behmer ST (1997). Importance of dietary nitrogen and carbohydrates to survival, growth, and reproduction in adults of the grasshopper *Ageneotettix deorum* (Orthoptera: Acrididae). Oecologia.

[72] Lee KP, Simpson SJ, Clissold FJ, Brooks R, Ballard JW, Taylor PW, Soran N, Raubenheimer D (2008). Lifespan and reproduction in *Drosophila*: new insights from nutritional geometry. PNAS.

[73] Roeder KA, Behmer ST, Davidowitz G (2014). Lifetime consequences of food protein-carbohydrate content for an insect herbivore. Funct Ecol.

[74] Behmer ST (2009). Insect herbivore nutrient regulation. Annu Rev Entomol.

[75] Finglas P, Roe M, Pinchen H, Berry R, Church S, Dodha S, Farron Wilson M, Swan G (2015). McCance and Widdowson’s the composition of foods.

[76] Raubenheimer D, Simpson S (2003). Nutrient balancing in grasshoppers: behavioural and physiological correlates of dietary breadth. J Exp Biol.

[77] Zwolfer H, Cavalloro R. Life systems and strategies of resource exploitation in tephritids. In: Proceedings of the CEC/IOBC international symposium on fruit flies of economic importance, Athens, Greece, 16–19 November 1982. Rotterdam: AA Balkema, Published; 1983. p. 16–30.

[78] Renwick JAA (2001). Variable diets and changing taste in plant–insect relationships. J Chem Ecol.

[79] Bateman M (1972). The ecology of fruit flies. Annu Rev Entomol.

[80] Erbout N, De Meyer M, Vangestel C, Lens L (2009). Host plant toxicity affects developmental rates in a polyphagous fruit fly: experimental evidence. Biol J Linnean Soc.

[81] Ali JG, Agrawal AA (2012). Specialist versus generalist insect herbivores and plant defense. Trends Plant Sci.

[82] Balagawi S, Vijaysegaran S, Drew RA, Raghu S (2005). Influence of fruit traits on oviposition preference and offspring performance of *Bactrocera tryoni* (Froggatt) (Diptera: tephritidae) on three tomato (*Lycopersicon lycopersicum*) cultivars. Aust J Entomol.

[83] Díaz-Fleischer F, Aluja M (2003). Clutch size in frugivorous insects as a function of host firmness: the case of the tephritid fly *Anastrepha luden*s. Ecol Entomol.

[84] Greany P, Styer S, Davis P, Shaw P, Chambers D (1983). Biochemical resistance of citrus to fruit flies. Demonstration and elucidation of resistance to the Caribbean fruit fly *Anastrepha suspensa*. Entomol Exp Appl.

[85] Papachristos DP, Papadopoulos NT, Nanos GD (2008). Survival and development of immature stages of the Mediterranean fruit fly (Diptera: Tephritidae) in citrus fruit. J Econ Entomol.

[86] Papachristos DP, Papadopoulos NT (2009). Are citrus species favorable hosts for the Mediterranean fruit fly? A demographic perspective. Entomol Exp Appl..

[87] Smirle M (1993). Larval performance of two leafroller species on known and potential hosts. Entomol Exp Appl.

