# Supplementary material for: Host plant range of a fruit fly community (Diptera: Tephritidae): does fruit composition influence larval performance?
Source: BMC Ecol. 2016 Sep 20;16:40. doi: 10.1186/s12898-016-0094-8 (PMC5030732; doi:10.1186/s12898-016-0094-8)
Supplement: Supplementary file 1 — 10.1186/s12898-016-0094-8 Nutrient contents obtained from the literature for 22 fruit species (g or mg per 100 g of pulp). [file 12898_2016_94_MOESM1_ESM.docx]

Host plant range of a fruit fly community (Diptera: Tephritidae): Does fruit composition influence larval performance?

Hafsi Abir^1,2^, Facon Benoit^3^, Ravigné Virginie^1^, Chiroleu Frédéric^1^, Quilici Serge^1^_,_ Chermiti Brahim^2^, & Duyck Pierre-François^1^

^1^ CIRAD, UMR PVBMT, F-97410 Saint Pierre, France

^2^ Institut Supérieur Agronomique de Chott-Mariem, laboratoire d’Entomologie et de Lutte Biologique, Université de Sousse, 4042, Sousse, Tunisie

^3^ UMR « Centre de Biologie pour la Gestion des Populations », INRA-SPE, 755 avenue du Campus, Agropolis, CS 30016, 34988 Montferrier sur Lez, Cedex, France

Corresponding author: Duyck Pierre-François: [pierre-francois.duyck@cirad.fr](mailto:pierre-francois.duyck@cirad.fr)

UMR « Peuplements Végétaux et Bio-agresseurs en Milieu Tropical », CIRAD Pôle de Protection des Plantes, 7 chemin de l’Irat, 97410 Saint Pierre, La Réunion, France

**Additional file 1**

**Table 1.** Nutrient contents obtained from the literature for 22 fruit species (g or mg per 100 g of pulp).

| Fruit | Nutrients | | | | | | | | | | | | References |
| --- | --- | --- | --- | --- | --- | --- | --- | --- | --- | --- | --- | --- | --- |
|  | Water | Protein | Glucide | Fat | Fiber | Na | Mg | P | K | Ca | Iron | Vitamin C |  |
|  | (g) | (g) | (g) | (g) | (g) | (mg) | (mg) | (mg) | (mg) | (mg) | (mg) | (mg) |  |
| Eggplant | 92.47 | 5.88 | 1.19 | 0.24 | 2.15 | 2.50 | 14.83 | 14 | 216 | 14 | 0.23 | 7.10 | [[1-4](#_ENREF_1)] |
| Indian almond | 60.83 | 1.42 | 1.94 | 1.11 | 3.10 | 1.95 | 1.26 | 3 | 23 | 1 | 0.73 | 0.37 | [[5-9](#_ENREF_5)] |
| Loquat | 86.80 | 12.07 | 0.45 | 0.23 | 1.61 | 1.50 | 13.67 | 25 | 244 | 13 | 0.25 | 2.27 | [[4](#_ENREF_4), [10-15](#_ENREF_10)] |
| Carambola | 91.01 | 5.21 | 0.83 | 0.31 | 2.29 | 2.60 | 10.62 | 14 | 150 | 5 | 0.26 | 30.13 | [[4](#_ENREF_4), [15-19](#_ENREF_15)] |
| Chayote | 92.62 | 3.91 | 0.91 | 0.17 | 1.20 | 2.00 | 14.00 | 18 | 125 | 16 | 0.37 | 7.70 | [[4](#_ENREF_4), [20](#_ENREF_20), [21](#_ENREF_21)] |
| Pumpkin | 88.99 | 5.93 | 1.68 | 0.28 | 0.66 | 1.00 | 12.00 | 44 | 340 | 21 | 0.80 | 12.00 | [[4](#_ENREF_4), [22-26](#_ENREF_22)] |
| Custard apple | 74.40 | 21.81 | 1.65 | 0.45 | 2.02 | 4.00 | 18.00 | 28 | 382 | 29 | 0.71 | 19.20 | [[4](#_ENREF_4), [27](#_ENREF_27)] |
| Cucumber | 96.21 | 1.89 | 0.56 | 0.18 | 0.67 | 3.13 | 20.01 | 21 | 145 | 14 | 0.20 | 3.47 | [[4](#_ENREF_4), [15](#_ENREF_15), [28](#_ENREF_28), [29](#_ENREF_29)] |
| Zucchini | 93.61 | 2.80 | 1.81 | 0.31 | 1.15 | 2.68 | 29.85 | 64 | 295 | 22 | 0.78 | 22.40 | [[4](#_ENREF_4), [30-33](#_ENREF_30)] |
| Fig | 80.43 | 15.16 | 1.02 | 0.77 | 2.25 | 4.28 | 35.09 | 15 | 434 | 105 | 0.43 | 2.00 | [[4](#_ENREF_4), [15](#_ENREF_15), [34-37](#_ENREF_34)] |
| Guava | 79.63 | 15.07 | 1.68 | 0.47 | 4.71 | 6.00 | 22.00 | 50 | 244 | 45 | 0.26 | 208.49 | [[4](#_ENREF_4), [15](#_ENREF_15), [38-41](#_ENREF_38)] |
| Strawberry guava | 80.66 | 17.36 | 0.58 | 0.60 | 5.40 | 37.00 | 17.00 | 27 | 292 | 21 | 0.66 | 37.00 | [[4](#_ENREF_4)] |
| Mango | 80.30 | 13.89 | 0.97 | 0.28 | 1.39 | 2.11 | 19.37 | 15 | 218 | 16 | 0.65 | 36.70 | [[4](#_ENREF_4), [42-46](#_ENREF_42)] |
| Melon | 91.08 | 6.32 | 0.98 | 0.16 | 0.77 | 18.31 | 14.70 | 11 | 269 | 11 | 0.34 | 24.26 | [[4](#_ENREF_4), [15](#_ENREF_15), [47](#_ENREF_47), [48](#_ENREF_48)] |
| Papaya | 88.39 | 8.98 | 0.58 | 0.31 | 1.60 | 5.56 | 21.78 | 13 | 211 | 23 | 0.37 | 55.93 | [[4](#_ENREF_4), [15](#_ENREF_15), [49-51](#_ENREF_49)] |
| Water melon | 91.64 | 7.48 | 0.60 | 0.13 | 0.40 | 0.76 | 10.87 | 11 | 134 | 5 | 0.28 | 8.27 | [[4](#_ENREF_4), [15](#_ENREF_15), [52](#_ENREF_52)] |
| Peach | 88.17 | 10.02 | 0.74 | 0.26 | 1.83 | 0.80 | 11.77 | 20 | 215 | 13 | 0.71 | 13.40 | [[4](#_ENREF_4), [15](#_ENREF_15), [53-55](#_ENREF_53)] |
| Chilli | 90.95 | 4.97 | 1.38 | 0.29 | 1.60 | 6.00 | 16.50 | 32 | 249 | 12 | 0.34 | 112.05 | [[4](#_ENREF_4), [56](#_ENREF_56), [57](#_ENREF_57)] |
| Plum | 82.82 | 10.50 | 0.76 | 0.28 | 1.90 | 1.03 | 7.17 | 21 | 216 | 5 | 0.31 | 7.45 | [[15](#_ENREF_15), [58](#_ENREF_58), [59](#_ENREF_59)] |
| Mandarin | 85.89 | 11.96 | 0.81 | 0.27 | 1.77 | 3.45 | 12.13 | 22 | 162 | 33 | 0.77 | 24.03 | [[15](#_ENREF_15), [60](#_ENREF_60), [61](#_ENREF_61)] |
| Tomato | 93.52 | 4.48 | 0.87 | 0.19 | 1.23 | 6.43 | 12.84 | 26 | 197 | 10 | 0.47 | 18.05 | [[4](#_ENREF_4), [15](#_ENREF_15), [62-65](#_ENREF_62)] |
| Tree tomato | 88.53 | 6.90 | 1.07 | 0.36 | 3.30 | 1.44 | 20.60 | 39 | 321 | 11 | 1.21 | 29.80 | [[4](#_ENREF_4), [15](#_ENREF_15), [66-68](#_ENREF_66)] |


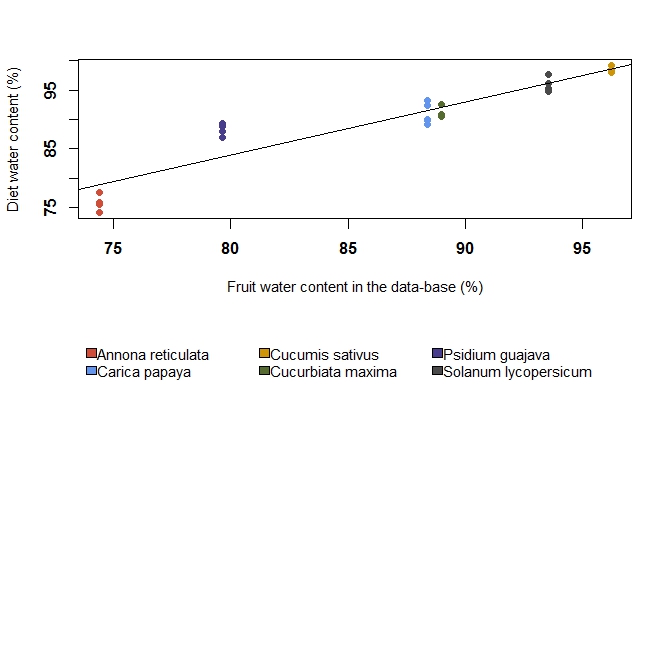


**Figure 1**. Relationship between diet water content and water content taken from database source for six fruit species (R²= 0.87; df_1, 28_= 191; P< 0.001;Y= 0.90 X + 12.13)


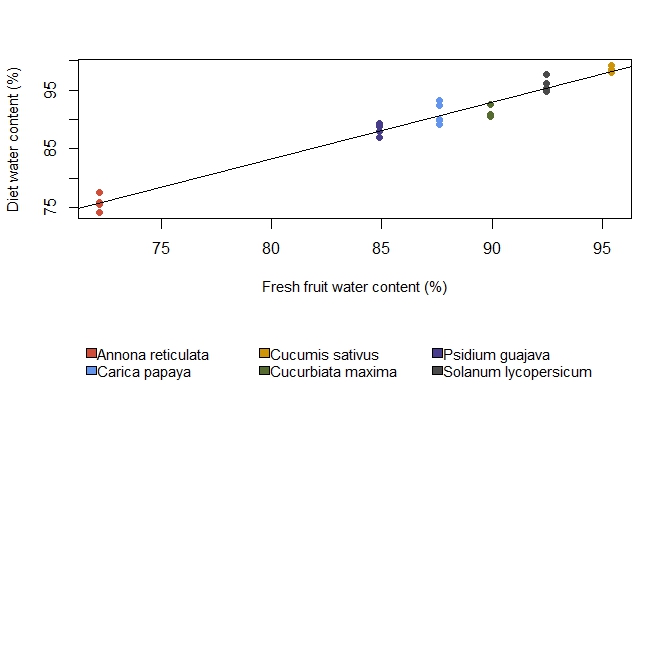


**Figure 2**. Relationship between diet water content and fresh fruit water content for six fruit species (R²= 0.97; df_1, 28_= 837.90; P< 0.001; Y= 0.96 X + 6.28)

**References**

1. Chen N, Li H. Cultivation and breeding of eggplant. In: *Training Workshop on Vegetable Cultivation and Seed Production: 1996*. Published.

2. Salunkhe DK, Kadam S. Handbook of vegetable science and technology: Production, compostion, storage, and processing. New York, USA: CRC press. 1998.

3. Sultana S, Iqbal A, Islam M. Preservation of carrot, green chilli and brinjal by fermentation and pickling. *Int Food Res J.* 2014; 21(6):2405-2412.

4. USDA. United States Department of Agriculture Agricultural Research Service: National Nutrient Database for Standard Reference. In*.*; 2015.

5. Oduro I, Larbie C, Amoako T, Antwi Boasiako A. Proximate composition and basic phytochemical assessment of two common varieties of *Terminalia catappa* (Indian Almond). *J Sci Technol (Ghana).* 2009; 29(2):1-6.

6. Marques MR, Paz DD, Batista LPR, Barbosa CDO, Araújo MAM, Moreira Araújo RSDR. An in vitro analysis of the total phenolic content, antioxidant power, physical, physicochemical, and chemical composition of *Terminalia Catappa* Linn fruits. *Food Sci Technol (Campinas).* 2012; 32(1):209-213.

7. Udotong JI, Bassey MI. Evaluation of the chemical composition, nutritive value and antinutrients of *Terminalia catappa* L. Fruit (Tropical Almond). *Int J En Tech Res.* 2015; 3:2454-4698.

8. Nwosu F, Dosumu O, Okocha J. The potential of *Terminalia catappa* (Almond) and *Hyphaene thebaica* (Dum palm) fruits as raw materials for livestock feed. *Afr J Biotechnol.* 2008; 7(24):4576-4580.

9. Dikshit M, Samudrasok RK. Nutritional evaluation of outer fleshy coat of *Terminalia catappa* fruit in two varieties. *Int J Food Sci Nutr.* 2011; 62(1):47-51.

10. Xu HX, Chen JW. Commercial quality, major bioactive compound content and antioxidant capacity of 12 cultivars of loquat (*Eriobotrya japonica* Lindl.) fruits. *J Sci Food Agric.* 2011; 91(6):1057-1063.

11. Abozeid WM, Nadir A. Physicochemical and organoleptic characteristics of loquat fruit and its processing. *Nat Sci.* 2012; 10:108-113.

12. Hasegawa PN, Faria AFd, Mercadante AZ, Chagas EA, Pio R, Lajolo FM, Cordenunsi BR, Purgatto E. Chemical composition of five loquat cultivars planted in Brazil. *Food Sci Technol (Campinas).* 2010; 30(2):552-559.

13. Vela JC, Marchart SS, Lucas IG, Martínez RB. A correlation study of loquat (*Eriobotrya japonica* cv. Algerie) fruit quality parameters: Flesh firmness and purple spotting. In: *First International Symposium on Loquat: 2002; Valencia, Spain*. Published 187-190.

14. Gariglio N, Agusti M. Effect of fruit thinning on the mineral composition of loquat (*Eriobotrya japonica* Lindl.) fruit and its connection with purple spot. *Span J Agric Res.* 2005; 3(4):439-445.

15. ANSES. Agence nationale de sécurité sanitaire de l’alimentation, de l’environnement et du travail :Table ciqual,https://pro.anses.fr/tableciqual/index.htm. In*.*; 2015.

16. Narain N, Bora P, Holschuh H, Vasconcelos MDS. Physical and chemical composition of carambola fruit (*Averrhoa carambola* l.) at three stages of maturity. *CYTA J Food.* 2001; 3(3):144-148.

17. Bhasker B, Shantaram M. Morphological and biochemical characteristics of *Averrhoa* fruits. *J Pharm Chem Biol Sci.* 2013; 3(3):924-928.

18. Soumya SL, Nair BR. Change in the biochemical profils of fruits of two species of averroa during development. *Int J Pharm Pharm Sci.* 2014; 6(4):572-577.

19. Patil AG, Patil DA, Phatak AV, Chandra N. Physical and chemical characteristics of carambola (*Averrhoa carambola* L.) fruit at three stages of maturity. *Int J Appl Biol Pharm.* 2010; 1(2):624-629.

20. Saade RL. Promoting the conservation and use of under utilistzed and neglected crops: Chayote *Sechium edule* (Jacq.) Sw, vol. 8. Rome, Italy: International plant genetic ressources institute. 1996.

21. Zinsou C, Sobesky O, Clairon M, Constant C. Composition minérale et glucidique du fruit de christophine ou chayote, *Sechium edule* Sw., au cours du grossissement du fruit. *Agronomie.* 1983; 3(6):529-536.

22. Muntean E, Muntean N, Duda MM. *Cucurbita maxima* Duch. as medicicnal plant. *Hop and Medicinal Plants.* 2014; 21(1-2):75-80.

23. Jacobo-Valenzuela N, de Jesus Zazueta-Morales J, Gallegos-Infante JA, Aguilar-Gutierrez F, Camacho-Hernandez IL, Rocha-Guzman NE, Gonzelez-Laredo RF. Chemical and physicochemical characterization of winter squash (*Cucurbita moschata* D.). *Not Bot Horti Agrobot Cluj Napoca.* 2011; 39(1):34-40.

24. Kim MY, Kim EJ, Kim YN, Choi C, Lee BH. Comparison of the chemical compositions and nutritive values of various pumpkin (Cucurbitaceae) species and parts. *Nutr Res Pract.* 2012; 6(1):21-27.

25. Nwofia GE, Nwogu NV, Nwofia BK. Nutritional variation in fruits and seeds of pumpkins (*Cucurbita Spp*) accessions from Nigeria. *Pak J Nutr.* 2012; 11(10):848-858.

26. Sharma S, Rao R. Nutritional quality characteristics of pumpkin fruit as revealed by its biochemical analysis. *Int Food Res J.* 2013; 20(5):2309-2316.

27. Pinto DQ, Cordeiro M, De Andrade S, Ferreira F, Filgueiras DC, Alves R, Kinpara D. Fruits for the future 5: Annona species. Southampton, UK. : International Centre for Underutilised Crops. 2005.

28. Belsito M, Hill RA, Klaassen CD, Liebler D, Marks Jr JG, Ronald C. Tentative safety assessment: *Cucumis Sativus* (Cucumber) derived Iingredients as used in cosmetics. Washington. 2012.

29. Gopalakrishnan SB, Kalaiarasi T. Comparative phytochemical screening of the fruits of *Cuculis trigonus* Roxb and *Cucumis sativus* Linn. *Int J Pharm Pharm Sci.* 2014; 3:1455-1468.

30. Karanja J, Mugendi J, Fathiya M, Muchugi A. Comparative study on the nutritional value of the pumpkin *Cucubuita maxima* varieties from defferent regions in Kenya. In: *Scientific Conference Proceedings: 2014*. Published.

31. Burkill HM. The useful plants of west tropical Africa, vol. 1. USA: Royal Botanic Gardens, Kew. 1995.

32. Eissa HA, Bareh GF, Ibrahim AA, Moawad RK, Ali HS. The effect of different drying methods on the nutrients and non-nutrients composition of zucchini (green squash) rings. *J Appl Sci Res.* 2013; 9(8):5380-5389.

33. Kmiecik W, Lisiewska Z. The influence of potassium sorbate addition on the quality of pickled zucchini fruit. *Rocz Panstw Zakl Hig.* 1993; 45(4):301-309.

34. Aljane F, Ferchichi A. Postharvest chemical properties and mineral contents of some fig (*Ficus carica* L.) cultivars in Tunisia. *J Food Agric Environ.* 2009; 7(2):209-212.

35. Nakilcioğlu E, Hışıl Y. Research on the phenolic compounds in Sarilop (*Ficus carica* L.) fig variety. *GIDA.* 2013; 38(5):267-274.

36. El-Shobaki F, El-Bahay A, Esmail R, El-Megeid A, Esmail N. Effect of figs fruit (*Ficus carica* L.) and its leaves on hyperglycemia in alloxan diabetic rats. *World J Diary food Sci.* 2010; 5(1):47-57.

37. Ficsor E, Szentmihályi K, Lemberkovics É, Blázovics A, Balázs A. Analyses of *Ficus carica* L. volatil components and mineral content. *Eur Chem Bull.* 2013; 2(3):126-129.

38. Wang F, Chen Y-H, Zhang Y-J, Deng G-F, Zou Z-F, Li A-N, Xu D-P, Li H-B. Chemical components and bioactivities of *Psidium guajava*. *Int J Food Nutr Saf.* 2014; 5(2):98-114.

39. Ruby J, Nathan P, Balasingh J, Kunz T. Chemical composition of fruits and leaves eaten by short-nosed fruit bat, *Cynopterus sphinx*. *J Chem Ecol.* 2000; 26(12):2825-2841.

40. Moreno MA, Zampini IC, Costamagna M, Sayago JE, Ordoñez RM, Isla MI. Phytochemical composition and antioxidant capacity of *Psidium guajava* fresh fruits and flour. *Food Nutr Sci.* 2014; 5(08):725.

41. Gull J, Sultana B, Anwar F, Naseer R, Ashraf M, Ashrafuzzaman M. Variation in antioxidant attributes at three ripening stages of guava (*Psidium guajava* L.) fruit from different geographical regions of Pakistan. *Mol.* 2012; 17(3):3165-3180.

42. Ubwa ST, Ishu MO, Offem JO, Tyohemba RL, Igbum GO. Proximate composition and some physical attributes of three mango (*Mangifera indica* L.) fruit varieties. *Int J Agro Agric Res.* 2014; 4(2):21-29.

43. Othman O, Mbogo G. Physico-chemical characteristics of storage-ripened mango (*Mangifera indica* L.) fruits varieties of eastern Tanzania. *Tanz J Sci.* 2009; 35(1):57-65.

44. Jahan S, Gosh T, Begum M, Saha B. Nutritional profile of some tropical fruits in Bangladesh: Specially anti-oxidant vitamins and minerals. *Bangladesh J Med Sci.* 2011; 10(2):95-103.

45. Gorinstein S, Poovarodom S, Leontowicz H, Leontowicz M, Namiesnik J, Vearasilp S, Haruenkit R, Ruamsuke P, Katrich E, Tashma Z. Antioxidant properties and bioactive constituents of some rare exotic Thai fruits and comparison with conventional fruits: *In vitro* and *in vivo* studies. *Int Food Res J.* 2011; 44(7):2222-2232.

46. Heath RR, Lavallee SG, Schnell E, Midgarden DG, Epsky ND. Laboratory and field cage studies on female-targeted attract-and-kill bait stations for Anastrepha suspensa (Diptera: Tephritidae). *Pest Manag Sci.* 2009; 65(6):672-677.

47. Haldhar SM, Bhargava R, Choudhary B, Pal G, Kumar S. Allelochemical resistance traits of muskmelon (*Cucumis melo*) against the fruit fly (*Bactrocera cucurbitae*) in a hot arid region of India. *Phytoparasitica.* 2013; 41(4):473-481.

48. Bello OO, Bello TK, Fashola MO, Oluwadun A. Microbiological quality of some locally produced fruit juices in Ogun state, South Western Nigeria. *J Microbiol Res.* 2014; 2(1):1-8.

49. Nwofia GE, Ojimelukwe P, Eji C. Chemical composition of leaves, fruit pulp and seeds in some *Carica papaya* (L) morphotypes. *Int J Med Arom Plants.* 2012; 2(1):200-206.

50. Aravind G, Debjit B, Duraivel S, Harish G. Traditional and medicinal uses of *Carica papaya*. *J Med Plants Stud.* 2013; 1(1):7-15.

51. Yogiraj V, Goyal PK, Chauhan CS, Goyal A, Vyas B. *Carica papaya* Linn: An overview. *Int J Herb Med.* 2014; 2(5):001-008.

52. Rahman B. Phytochemical investigation of *Citrullus lanatus* (Watermelon) rind. Dhaka, Bangladesh: East West University; 2013.

53. Ashraf C, Iqbal S, Ahmed D. Nutritional and physicochemical studies on fruit pulp, seed and shell of indigenous *Prunus persica*. *J Med Plants Res.* 2011; 5(16):3917-3921.

54. Gil MI, Tomás-Barberán FA, Hess-Pierce B, Kader AA. Antioxidant capacities, phenolic compounds, carotenoids, and vitamin C contents of nectarine, peach, and plum cultivars from California. *J Agric Food Chem.* 2002; 50(17):4976-4982.

55. Manzoor M, Anwar F, Mahmood Z, Rashid U, Ashraf M. Variation in minerals, phenolics and antioxidant activity of peel and pulp of different varieties of peach (*Prunus persica* L.) fruit from Pakistan. *Mol.* 2012; 17(6):6491-6506.

56. López-Hernández J, Oruña-Concha M, Simal-Lozano J, Vázquez-Blanco M, González-Castro M. Chemical composition of padrón peppers (*Capsicum annuum* L.) grown in Galicia (NW Spain). *Food Chem.* 1996; 57(4):557-559.

57. Simonovska J, Rafajlovska V, Kavrakovski Z, Srbinoska M. Nutritive and bioactive compounds in hot fruits of *Capsicum annuum* L. from Macedonia. *Maced J Chem Chem En.* 2014; 33(1):97-104.

58. Nergiz C, Yildiz H. Research on chemical composition of some varieties of European plums (*Prunus domestica*) adapted to the Aegean district of Turkey. *J Agric Food Chem.* 1997; 45(8):2820-2823.

59. Divya P, Pandey V. Natural antioxidants and phyto-chemicals in plant foods. India: Satish serial publishing house 2014.

60. Liu Y, Heying E, Tanumihardjo SA. History, global distribution, and nutritional importance of citrus fruits. *Comp Rev Food Sci Food Safety.* 2012; 11(6):530-545.

61. Boudries H, Madani K, Touati N, Souagui S, Medouni S, Chibane M. Pulp antioxidant activities, mineral contents and juice nutritional properties of Algerian clementine cultivars and mandarin. *Afr J Biotechnol.* 2014; 11(18):4285-4267.

62. Suárez MH, Rodríguez ER, Romero CD. Chemical composition of tomato (*Lycopersicon esculentum*) from Tenerife, the Canary Islands. *Food Chem.* 2008; 106(3):1046-1056.

63. Pinela J, Barros L, Carvalho AM, Ferreira IC. Nutritional composition and antioxidant activity of four tomato (*Lycopersicon esculentum* L.) farmer’varieties in Northeastern Portugal homegardens. *Food Chem Toxicol.* 2012; 50(3):829-834.

64. Sainju UM, Dris R, Singh B. Mineral nutrition of tomato. *J Food Agric Environ.* 2003; 1(2):176-184.

65. Zoran IS, Nikolaos K, Ljubomir Š. Tomato fruit quality from organic and conventional production:. In: *Organic agriculture towards sustainability.* Edited by Pilipavicius V. Rijeka, Croatia: Tech Europe. 2014; 147-169.

66. Nallakurumban P, Suja N, Vijayakumar A, Geetha PS, Karpagapandi L. Estimation of phytochemicals and antioxidant property of tamarillo (*Solanum betaceum*) and a value added product tamarillo Sauce. *Int J Sci Prog Res.* 2015; 9(2):61665.

67. Acosta Quezada PG, Raigon MD, Riofrio Cuenca T, Garcia Martinez MD, Plazas M, Burneo JI, Figueroa JG, Vilanova S, Prohens J. Diversity for chemical composition in a collection of different varietal types of tree tomato (*Solanum betaceum* Cav.), an Andean exotic fruit. *Food Chem.* 2015; 169:327-335.

68. Torres A. Physical, chemical and bioactive compounds of tree tomato (*Cyphomandra betacea*). *Arch Latinoam Nutr.* 2012; 62(4):381-388.
