# Supplementary material for: Host plant range of a fruit fly community (Diptera: Tephritidae): does fruit composition influence larval performance?
Source: BMC Ecol. 2016 Sep 20;16:40. doi: 10.1186/s12898-016-0094-8 (PMC5030732; doi:10.1186/s12898-016-0094-8)
Supplement: Supplementary file 2 — 10.1186/s12898-016-0094-8 CCA analysis of the relationship between fruit composition and survival of the larvae of seven tephritid species. (a) Position of the fruit species in the factorial space of CCA analyses with contribution of nutrient composition and larval survival on the studied fruits. (b) Contribution of the nutrient composition to the factorial space. (c) Position of larval survival of the seven tephritid species in the factorial space. Host fruits belonging to the Cucurbitaceae and Solanaceae families are in green and red, respectively. Ccat: C. catoirii, Ccap: C. capitata, Cros: C. rosa, Bzon: B. zonata, Zcuc : Z. cucurbitae, Ddem: D. demmerezi, Ncya: N. cyanescens. [file 12898_2016_94_MOESM2_ESM.docx]

Host plant range of a fruit fly community (Diptera: Tephritidae): Does fruit composition influence larval performance?

Hafsi Abir^1,2^, Facon Benoit^1,3^, Ravigné Virginie^1^, Chiroleu Frédéric^1^, Quilici Serge^1^_,_ Chermiti Brahim^2^, & Duyck Pierre-François^1^

^1^ CIRAD, UMR PVBMT, F-97410 Saint Pierre, France

^2^ Institut Supérieur Agronomique de Chott-Mariem, laboratoire d’Entomologie et de Lutte Biologique, Université de Sousse, 4042, Sousse, Tunisie

^3^ UMR « Centre de Biologie pour la Gestion des Populations », INRA-SPE, 755 avenue du Campus, Agropolis, CS 30016, 34988 Montferrier sur Lez, Cedex, France

Short title: Fruit fly host range and fruit composition

Corresponding author: Duyck Pierre-François: [pierre-francois.duyck@cirad.fr](mailto:pierre-francois.duyck@cirad.fr)

UMR « Peuplements Végétaux et Bio-agresseurs en Milieu Tropical », CIRAD Pôle de Protection des Plantes, 7 chemin de l’Irat, 97410 Saint Pierre, La Réunion, France

**Additional file 2**

CCA analysis of the relationship between fruit composition and survival of the larvae of seven tephritid species. (a) Position of the fruit species in the factorial space of CCA analyses with contribution of nutrient composition and larval survival on the studied fruits. (b) Contribution of the nutrient composition to the factorial space. (c) Position of larval survival of the seven tephritid species in the factorial space. Host fruits belonging to the Cucurbitaceae and Solanaceae families are in green and red, respectively. Ccat: *C. catoirii*, Ccap: *C. capitata*, Cros: *C. rosa*, Bzon: *B. zonata*, Zcuc : *Z. cucurbitae*, Ddem: *D. demmerezi*, Ncya: *N. cyanescens.*

^^
