# Supplementary material for: Host plant range of a fruit fly community (Diptera: Tephritidae): does fruit composition influence larval performance?
Source: BMC Ecol. 2016 Sep 20;16:40. doi: 10.1186/s12898-016-0094-8 (PMC5030732; doi:10.1186/s12898-016-0094-8)
Supplement: Supplementary file 3 — 10.1186/s12898-016-0094-8 Duration (days) of the larval stage of seven tephritid species reared on 22 different host fruits occurring in La Reunion. Values are means ± SE. Host fruits belonging to the Cucurbitaceae and Solanaceae families are in green and red, respectively. Host fruits are ordered by coordinate of the first axis of the CCA analyses (see “Methods” section). [file 12898_2016_94_MOESM3_ESM.docx]

Host plant range of a fruit fly community (Diptera: Tephritidae): Does fruit composition influence larval performance?

Hafsi Abir^1,2^, Facon Benoit^1,3^, Ravigné Virginie^1^, Chiroleu Frédéric^1^, Quilici Serge^1^_,_ Chermiti Brahim^2^, & Duyck Pierre-François^1^

^1^ CIRAD, UMR PVBMT, F-97410 Saint Pierre, France

^2^ Institut Supérieur Agronomique de Chott-Mariem, laboratoire d’Entomologie et de Lutte Biologique, Université de Sousse, 4042, Sousse, Tunisie

^3^ UMR « Centre de Biologie pour la Gestion des Populations », INRA-SPE, 755 avenue du Campus, Agropolis, CS 30016, 34988 Montferrier sur Lez, Cedex, France

Corresponding author: Duyck Pierre-François: [pierre-francois.duyck@cirad.fr](mailto:pierre-francois.duyck@cirad.fr)

UMR « Peuplements Végétaux et Bio-agresseurs en Milieu Tropical », CIRAD Pôle de Protection des Plantes, 7 chemin de l’Irat, 97410 Saint Pierre, La Réunion, France

**Additional file 3**

Duration (days) of the larval stage of seven tephritid species reared on 22 different host fruits occurring in La Reunion. Values are means ± SE. Host fruits belonging to the Cucurbitaceae and Solanaceae families are in green and red, respectively. Host fruits are ordered by coordinate of the first axis of the CCA analyses (see Materials and Methods)**.**

^
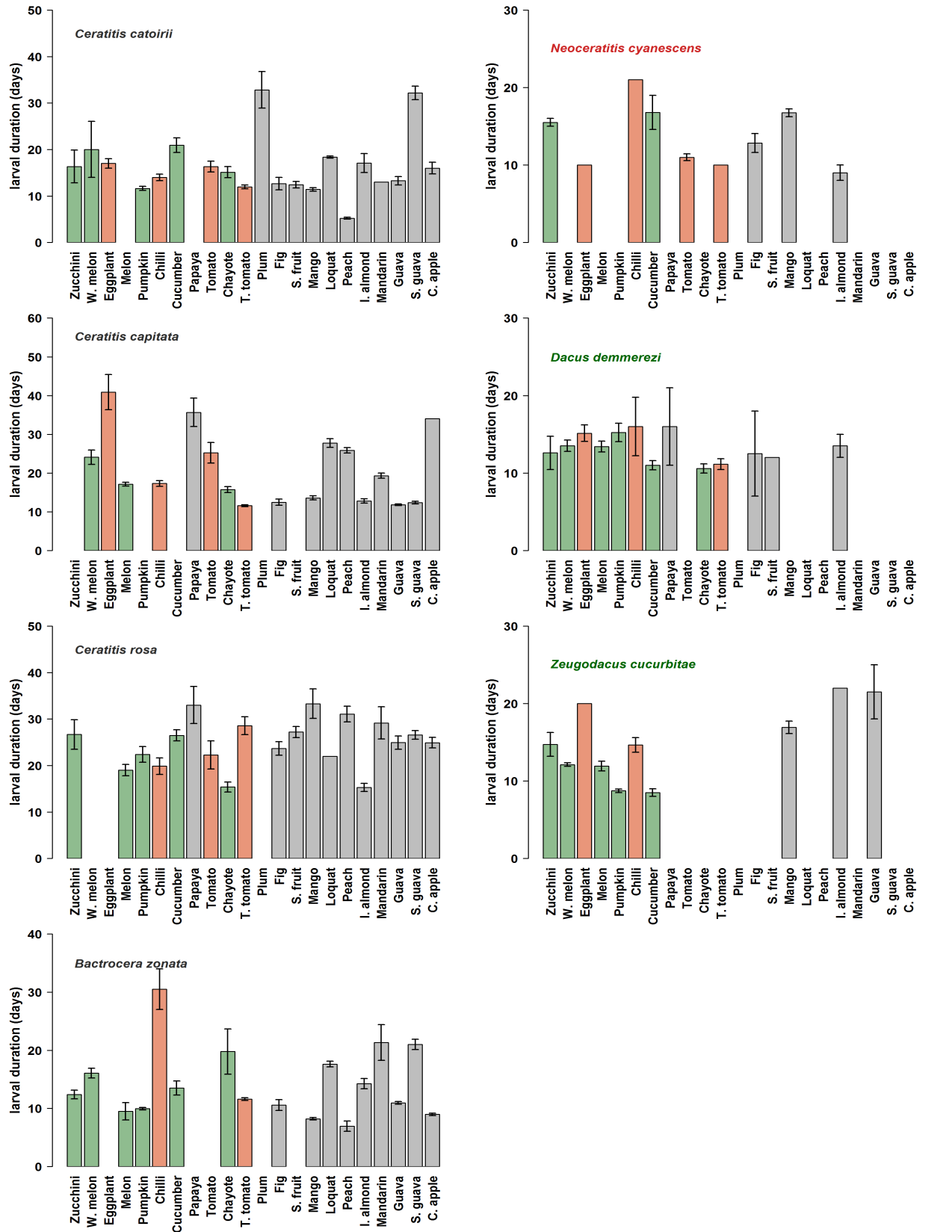
^
