# Supplementary material for: Host plant range of a fruit fly community (Diptera: Tephritidae): does fruit composition influence larval performance?
Source: BMC Ecol. 2016 Sep 20;16:40. doi: 10.1186/s12898-016-0094-8 (PMC5030732; doi:10.1186/s12898-016-0094-8)
Supplement: Supplementary file 4 — 10.1186/s12898-016-0094-8 Relationship between pupal weight (10−4 g) and larval duration (days) for seven tephritid species reared on 22 different host fruits. Determination coefficients (R2) and regression lines are given when relationships are significant (P< 0.05). [file 12898_2016_94_MOESM4_ESM.docx]

Host plant range of a fruit fly community (Diptera: Tephritidae): Does fruit composition influence larval performance?

Hafsi Abir^1,2^, Facon Benoit^3^, Ravigné Virginie^1^, Chiroleu Frédéric^1^, Quilici Serge^1^_,_ Chermiti Brahim^2^, & Duyck Pierre-François^1^

^1^ CIRAD, UMR PVBMT, F-97410 Saint Pierre, France

^2^ Institut Supérieur Agronomique de Chott-Mariem, laboratoire d’Entomologie et de Lutte Biologique, Université de Sousse, 4042, Sousse, Tunisie

^3^ UMR « Centre de Biologie pour la Gestion des Populations », INRA-SPE, 755 avenue du Campus, Agropolis, CS 30016, 34988 Montferrier sur Lez, Cedex, France

Corresponding author: Duyck Pierre-François: [pierre-francois.duyck@cirad.fr](mailto:pierre-francois.duyck@cirad.fr)

UMR « Peuplements Végétaux et Bio-agresseurs en Milieu Tropical », CIRAD Pôle de Protection des Plantes, 7 chemin de l’Irat, 97410 Saint Pierre, La Réunion, France

**Additional file 4**

Relationship between pupal weight (10^–4^ g) and larval duration (days) for seven tephritid species reared on 22 different host fruits. Determination coefficients (R²) and regression lines are given when relationships are significant (P< 0.05).

^^
